# Diagnosis and Challenges in Perinatal Health

**DOI:** 10.3390/diagnostics12061399

**Published:** 2022-06-06

**Authors:** Blanca Riquelme-Gallego, Rafael A. Caparros-Gonzalez

**Affiliations:** 1 Department of Nursing, Faculty of Health Sciences, Universidad de Granada, Avenida de la Ilustración nº 60 C.P., 18071 Granada, Spain; rcg477@ugr.es; 2 Instituto de Investigación Biosanitaria (ibs, GRANADA), 18014 Granada, Spain

Perinatal health is a primary objective for health systems. The perinatal period is a sensitive time for both the mother and the fetus. During pregnancy, delivery, and the postpartum period, numerous physiological and psychological changes take place that can affect maternal and neonatal health. Therefore, there are numerous situations that may have an adverse impact during the perinatal period, which need to be assessed and diagnosed early [1]. 

Prenatal diagnosis refers to the set of tests that are performed during pregnancy in order to identify the presence of potential congenital defects in the fetus or maternal risk factors that may require strict controls throughout gestation. The early diagnosis of any congenital defect in the fetus allows the most appropriate measures to be taken, both during pregnancy and during delivery, to prevent unnecessary risks for the mother and child and to try to improve the prognosis of the newborn after birth [2]. In some cases, intrauterine treatment of certain congenital defects can nowadays be carried out. Likewise, in the presence of certain fetal pathologies, preparation for receiving the newborn by a multidisciplinary team can improve the postnatal prognosis of the newborn [3].

There are a series of conditions that increase the possibility of having a child with congenital defects or intrauterine growth disorders. These conditions or risk factors are the reason why, in addition to routine screening tests, it is advisable to carry out specific tests on certain pregnant women. Pregnant women meeting one or more of the following conditions should be considered as a high-risk pregnancy: a previous child with chromosomal abnormalities or other congenital defects, mother or father with a chromosomal abnormality, suffering from an X-linked disease, having a chronic disease such as diabetes or certain endocrine disorders, having a family history of malformations, chromosomal abnormalities or congenital metabolic disorders, suffering from an infection during pregnancy, twin pregnancy, maternal obesity, advanced maternal age (>35 years old), consuming drugs during pregnancy, being exposed to radiation or toxic products for work-related or temporary reasons, assisted reproductive techniques. The possibility of a pregnant woman with one or more of these conditions or having a fetus with a congenital defect is multiplied by a greater or lesser factor that depends on the altered condition detected [1,4,5,6]. Additionally, suffering from a mental health issue during the perinatal period (i.e., stress, anxiety, depression, psychopathological symptoms, post-traumatic stress disorder) is a risk factor that can have a negative impact on the maternal health and the offspring [7].

A distinction should be made between “screening techniques” which only allow the assessment of a “risk index” for certain anomalies and “diagnostic techniques” capable of reliably identifying the congenital defect. They are also known as “screening” techniques, and their aim is to identify pregnancies that, although not associated with the risk factors mentioned, present a higher than expected “risk index”. The detection, by any of the procedures mentioned here, of a high risk of congenital defects makes it advisable to carry out a diagnostic test [8,9,10].

Why is it necessary to carry out “universal” screening techniques for chromosomal anomalies in the entire population? Firstly, because it is not justified to perform a diagnostic test on all pregnant women as a first option due to the risks involved; secondly, because of the high cost in terms of economic and health care resources involved; and thirdly, because a well-conducted screening will allow us to identify a subgroup of patients who will really benefit from an invasive test.

There are different screenings from which this most vulnerable subgroup can benefit: EBA screening, which detects about 90% of all chromosomal abnormalities; advanced screening by detecting fetal DNA in maternal blood; screening for risk of pre-eclampsia, among others [8].

Regarding prenatal diagnostic techniques, there are numerous tests, many of which are included in many health systems. The choice of the most appropriate one depends on the personal circumstances of the pregnant woman, the stage of pregnancy, and the congenital defect type to be identified. Some of them are the detection of chromosomal abnormalities through chorionic villus sampling and amniocentesis, the detection of inherited genetic diseases such as cystic fibrosis, metabolic diseases, or muscular dystrophies; the detection of fetal malformations or infections such as toxoplasmosis or rubella infections, among others [11,12,13,14,15,16].

Maternal and child health professionals need to be trained in the detection and screening of these potential pregnancy complications, as pregnancy is an excellent opportunity to detect these problems and their risk factors. This process requires an individualized approach, the involvement of the women concerned, and the up-to-date knowledge of professionals about the associated risk factors. In summary, the best intervention is to detect and prevent pregnancy complications and to help achieve satisfactory obstetric-perinatal outcomes.
